# Multidisciplinary studies on a sick-leader syndrome-associated mass stranding of sperm whales (*Physeter macrocephalus*) along the Adriatic coast of Italy

**DOI:** 10.1038/s41598-018-29966-7

**Published:** 2018-08-01

**Authors:** Sandro Mazzariol, Cinzia Centelleghe, Bruno Cozzi, Michele Povinelli, Federica Marcer, Nicola Ferri, Gabriella Di Francesco, Pietro Badagliacca, Francesca Profeta, Vincenzo Olivieri, Sergio Guccione, Cristiano Cocumelli, Giuliana Terracciano, Pasquale Troiano, Matteo Beverelli, Fulvio Garibaldi, Michela Podestà, Letizia Marsili, Maria Cristina Fossi, Simonetta Mattiucci, Paolo Cipriani, Daniele De Nurra, Annalisa Zaccaroni, Silva Rubini, Daniela Berto, Yara Beraldo de Quiros, Antonio Fernandez, Maria Morell, Federica Giorda, Alessandra Pautasso, Paola Modesto, Cristina Casalone, Giovanni Di Guardo

**Affiliations:** 1 Department of Comparative Biomedicine and Food Science, University of Padova, Padova, Italy; 2 Department of Animal Medicine, Production and Health, University of Padova, Padova, Italy; 3 Istituto Zooprofilattico Sperimentale dell’Abruzzo e del Molise “G. Caporale”, Teramo, Italy; 4 University of Teramo, Faculty of Veterinary Medicine, Località Piano d’Accio, 64100 Teramo, Italy; 5 Centro Studi Cetacei, Pescara (CSC), Italy; 6 Istituto Zooprofilattico Sperimentale del Lazio e della Toscana M. Aleandri, Rome, Italy; 7 Istituto Zooprofilattico Sperimentale della Puglia e della Basilicata, Foggia, Italy; 8 Department DISTAV, University of Genova, Genova, Italy; 9 Museum of Natural History of Milan, Milano, Italy; 10 Department of Physical Science, Earth and Environment, University of Siena, Siena, Italy; 11 Department of Public Health and Infectious Diseases, University La Sapienza, Rome, Italy; 12 Istituto Zooprofilattico Sperimentale della Sardegna, Sassari, Italy; 13 Department of Veterinary Science, University of Bologna, Bologna, Italy; 14 Istituto Zooprofilattico Sperimentale della Lombardia e dell’Emilia Romagna, Ferrara, Italy; 15 ISPRA, Chioggia, Italy; 16 Institute of Animal Health and Food Safety, Universitad de Las Palmas de Gran Canaria, Las Palmas, Spain; 17 Institute for Neurosciences of Montpellier (Inserm UMR 1051), Montpellier, France; 18 Istituto Zooprofilattico Sperimentale del Piemonte, Liguria e Val d’Aosta, Torino, Italy

## Abstract

Mass strandings of sperm whales (*Physeter macrocephalus*) are rare in the Mediterranean Sea. Nevertheless, in 2014 a pod of 7 specimens stranded alive along the Italian coast of the Central Adriatic Sea: 3 individuals died on the beach after a few hours due to internal damages induced by prolonged recumbency; the remaining 4 whales were refloated after great efforts. All the dead animals were genetically related females; one was pregnant. All the animals were infected by dolphin morbillivirus (DMV) and the pregnant whale was also affected by a severe nephropathy due to a large kidney stone. Other analyses ruled out other possible relevant factors related to weather conditions or human activities. The results of multidisciplinary *post-mortem* analyses revealed that the 7 sperm whales entered the Adriatic Sea encountering adverse weather conditions and then kept heading northward following the pregnant but sick leader of the pod, thereby reaching the stranding site. DMV infection most likely played a crucial role in impairing the health condition and orientation abilities of the whales. They did not steer back towards deeper waters, but eventually stranded along the Central Adriatic Sea coastline, a real trap for sperm whales.

## Introduction

In the last decades, cetacean mass strandings have attracted a growing attention from the media: these dramatic events induced an increase of the public concern towards the role of human activities and their effects on marine mammal conservation, with special emphasis on those related to anthropogenic sound sources. In particular, the use of naval sonar has been associated with both behavioral and pathological changes leading to lethal mass stranding of several cetacean species^1–3^. Sonar is not the only known, anthropogenic sound-related factor in the oceans associated to these dramatic events: other military and industrial activities like seismic surveys (Madagascar, 2008) have been also linked to the phenomenon^4^. A study conducted by Madsen and colleagues^5^ has also suggested that acoustic disturbance due to the presence of wind turbines in the underwater environment could affect the behavior of marine mammals, similarly to naval sonar.

However, mass strandings do not exclusively result from interaction with anthropic activities. Several natural factors are also considered equally responsible for similar events, including anomalies of the Earth geomagnetic field due to solar activity^6,7^, or the effects of lunar cycles^8^, meteorological and oceanographic factors like large-scale climatic events^9^, local disturbances and basin-related temperature variations that may influence prey distribution^10^. Furthermore, specific geographical and physical features of the coastlines could affect acoustic reflection and impair orientation and perception: these factors may influence mass strandings and thus explain the aggregation of such events in specific areas^11^. The Adriatic Sea is one of these “*cul-de-sacs*”, potentially fatal for sperm whales (*Physeter macrocephalus*)^12^: in fact, despite this species not being considered a regular inhabitant of this narrow arm of the Mediterranean Sea^13^, 7 different mass stranding events have occurred since 1555^14^, the latest of which in September 2014.

The present work reports the *post-mortem* findings obtained from the sperm whales involved in this last stranding episode, thereby proposing a reasonable hypothesis based on our multidisciplinary studies, literature and previous experience^12,15^.

## Results

A pod of 5 sperm whales was reported in Croatian waters by the Blue World Institute near the Vis Island (September 7, 2014) and later close to the Kornati Archipelagos (September 9, 2014). This sighting was communicated to the Italian Stranding Network, already experienced by a previous sperm whale mass stranding event occurred in 2009 along the Southern Adriatic coast of Italy. Consequently, the response was rapid when the 7 sperm whales were found beached alive on September 11th, early in the morning at Punta Penna beach, Nature reserve Punta Aderci, Vasto, Abruzzo Adriatic coastline (coordinates 42°10′28.0-N 14°42′02.4-E). The whales codenamed SW1, SW2 and SW3 did not survive the stranding. However, thanks to the efficient efforts of hundreds of volunteers coordinated by the personnel affiliated to the Centro Studi Cetacei Onlus, 4 animals were successfully refloated (https://www.youtube.com/watch?v=jExOUfEb1kc&t=279s). The outcome of refloating operation cannot be determined, as the released whales found the way to the high seas and were not seen again. However, a sperm whale carcass was found 14 days later along the Italian coast, about 130 nautical miles southward. Unfortunately, it cannot be assumed or excluded that the carcass belonged to one of the refloated specimens, since no further analysis was performed due to the advanced stage of decomposition.

The databases consulted (see Methods) reported no anomalous weather and/or marine condition, no anomalies of the solar and lunar cycles or changes in the geomagnetic field. Our current opinion is that the stranding site was determined by the sum of different environmental co-factors, namely wind, waves and marine currents. No official military exercises nor seismic surveys were ongoing in the Adriatic Sea during the stranding events or in the previous days.

### Biology

The whale pod consisted of 2 adult females (SW1 and SW2), a juvenile female (SW3), and 4 unsexed individuals. Data regarding total length, gender and age estimation, conservation code^16^ and nutritional conditions, either evaluated during necropsies (SW1, SW2 and SW3) or estimated during release efforts (SW4, SW5, SW6 and SW7), are reported in Table 1. The oldest animal (SW1) was an adult, sexually active animal, as confirmed by the pregnancy found during necropsy. SW2 also had functional gonads (see below).

**Table 1:** Biological data and conservation code of stranded animals: weight was estimated using Lockyer *et al*.^67^ on total length measured on the field.

SW	Total length	Estimated weight^16^	Conservation code^16^	Nutritional status	Estimated Age^16^	Sex
1	895 cm	8.84 t	2	2	31–32 years	F
1b	98 cm	n.e.	2	n.e.	n.e.	M
2	838 cm	7.38 t	3	2	21 years	F
3	733 cm	5,11 t	2	2	14 years	F
4*	650 cm	3,7 t	1	n.e.	juvenile	n.e.
5*	700 cm	4,5 t	1	n.e.	juvenile	n.e.
6*	750 cm	5,5 t	1	n.e.	juvenile	n.e.
7*	620 cm	3,2 t	1	n.e.	juvenile	n.e.

Sex, nutritional status and conservation code were assessed by visual evaluation. Age was estimated on teeth analyses on examined animals or estimated on total length.

*Data reported for these animals were estimated during refloation. n.e. means not evaluated.

Unfortunately, comparison of pictures of the stranded animals with photo-identification catalogues of sperm whales existing in the Mediterranean Sea (see Methods) showed no positive match.

### Genetic analyses

No genetic variability was detected in the mtDNA region of the analyzed samples. The haplotype detected in the mitochondrial control region of all samples was found to be the one reported in sperm whales of the Mediterranean Sea (haplotype 1, according to Drouot and colleagues^17^).

Descriptive parameters of genetic diversity calculated using 16 STRs demonstrated that our samples conserved a good genetic variability. All loci were in Hardy-Weinberg equilibrium. All markers selected for the analysis, except three (GATA02; GATA41; MK6), demonstrated a good informativeness; therefore, the selected panel of STR markers showed adequate discrimination ability for this study.

The genotypes were clustered by FCA and the graphical representation of the distribution of genetic distances between individuals showed that the samples were divided into two distinct clusters: the group including samples from the Mediterranean Marine Mammal Tissue Bank of the University of Padova, and that of the sperm whales included in the Vasto mass stranding event here reported (Supplementary Figure S1).

### Parentage analysis

#### Best cluster

The analyses conducted with different parameters converged to the same results. Best cluster output according to the method implemented in the COLONY software showed four genetic clusters, as reported in Table 2. Offspring sharing the same father ID (no matter whether the father is found in the collected samples or not) are paternal sibs, those sharing the same mother ID are maternal sibs.

**Table 2:** Results of kinship analysis by COLONY software: four clusters are identified, candidate Father and Mother ID are reported.

ClusterIndex	OffspringID	FatherID	MotherID
1	SWA	*1	#1
1	SWD	*1	#4
2	SWB	*2	#2
3	SWC	*3	#3
4	SW1	*4	#5
4	SW2	*4	#6
4	SW3	*4	#6

When the inferred father /mother is not found among the samples, the father /mother ID is given an index (starting from 1) prefixed with “*” and “#” for mother.

Our results indicate that the samples provided by the MMMTB of the University of Padova form three different clusters. Samples from whales SWA and SWB belong to the same cluster and share the candidate father n. 1 (they are paternal sibs). Samples SWB and SWC give rise to two separate clusters and do not share any parents. The samples from the Vasto mass stranding belong to a single cluster and result paternal sibs sharing the same candidate father (n. 4). Moreover, samples from SW2 and SW3 resulted full sib, indicating that the two whales share both the candidate father n. 4 and the candidate mother n. 6.

Based on the genotypes supplied, the software calculated the probability of all full sib dyads considering four possible pairs (Table 2). Only the SW2 and SW3 samples showed a high probability (94%) as full-sibs, confirming the results of best cluster analysis. Full sib-ship is difficult to infer reliably in our conditions, considering the small dataset and impossibility to determine with certainty parent-offspring relationships. Therefore, some parent-offspring relationships may be mistakenly inferred to be full-sib. However, the probability of the full-sib dyad here is rather high, since a) the data is confirmed in the best cluster results; and b) the age of the two subjects (21 and 14 years, respectively) lends support to the hypothesis of the two whales being full sisters.

**Table 3:** Stable isotope analysis of carbon and nitrogen in different tissues from the 3 stranded sperm whales under study.

	N	Mean	Minimum	Maximum	Std.Dev.
**Skin**					
δ_13_C ‰	3	−18.79	−19.73	−17.67	1.04
δ_13_C lipid free‰	3	−15.90	−16.66	−15.22	0.72
δ_15_N‰	3	10.07	9.80	10.31	0.26
δ_15_N lipid estratti ‰	3	10.30	10.03	10.59	0.28
C/N (mol/mol)	3	9.65	5.48	14.90	4.81
**Blubber**					
δ_13_C ‰	3	−19.37	−19.54	−19.07	0.26
δ_13_C lipid free‰	3	−15.90	−16.66	−15.22	0.72
δ_15_N‰	3	11.37	10.48	12.27	0.89
δ_15_N lipid free‰	3	11.46	11.08	11.94	0.44
C/N (mol/mol)	3	14.97	9.28	23.87	7.80
**Muscle**					
δ_13_C ‰	3	−16.43	−16.78	−16.11	0.34
δ_13_C lipid estratti ‰	3	−15.99	−16.48	−15.71	0.43
δ_15_N‰	3	8.90	8.58	9.36	0.40
δ_15_N lipid estratti ‰	3	9.59	9.08	10.05	0.49
C/N (mol/mol)	3	5.01	4.65	5.53	0.46
**Liver**					
δ_13_C ‰	3	−17.22	−17.89	−16.68	0.62
δ_13_C lipid free‰	3	−16.24	−16.52	−16.06	0.24
δ_15_N‰	3	8.89	8.76	8.97	0.11
δ_15_Nlipid free‰	3	9.52	9.37	9.78	0.23
C/N (mol/mol)	3	4.65	4.40	4.86	0.23

#### Population genetic structure

The Bayesian clustering analysis implemented in the STRUCTURE software indicated that the Ln probability of the genetic data, consistent among the five runs, was highest for k ≤ 2. The result agrees with the outcome of analysis according to Evanno *et al*.^18^, K = 2. Providing information on the sampling location did not influence the outcome of the analysis. Our results showed that the investigated samples were differentiated into two subpopulations, as specified here below: subpopulation A: SWA, SWB, SWC and SWD samples; subpopulation B: SW1, SW2 and SW3 samples.

The measure of differentiation between the two populations due to genetic structure (F*st*) was calculated according to Weir & Cockerham^19^ by FSTAT Software version 2.9.3.2^20^. The F*st* value was 0.03400, with a *p*-value < 0.05, which suggests a limited genetic differentiation.

### Stable isotope analyses

The carbon/nitrogen molar ratios (C:N) in the liver and muscle were similar for the 3 sperm whales. On the other hand, whale SW3 showed skin and blubber values higher than SW1 and SW2 (Table 3). Mean δ^15^N values in the 3 sperm whales showed an isotopic fractionation (ranged by only 1‰) for skin and blubber, as compared to skeletal muscle and liver tissues, which showed analogous values. Similar data were also obtained for δ^13^C values, showing less enrichment in ^13^C in the blubber and skin in comparison to the other tissues. The values of δ^13^C analysis of lipids extracted from the blubber (−19.37 ± 0.26‰) showed a depletion of δ^13^C (δ^13^Cbulk- δ^13^Clipid free) ranging at ∼3.4 in relation to the respective tissues. The variability of δ^13^C was lower in the liver (0.98) and muscle (0.44), considering that these tissues contain minimal amounts of lipids (low C/N values). The δ^15^N values conflicting results recommend caution in the δ^15^N correction. The variability reported in this study was <0.7‰.

### Pathological findings

The bodies of SW1–3 showed manifest changes related to prolonged recumbency in shallow waters, including diffuse visceral congestion, subcutaneous and subpleural hemorrhagic foci, pulmonary edema, mild peritoneal and/or pleural effusion, pneumo-mediastinum and lung emphysema.

**Figure 1:**
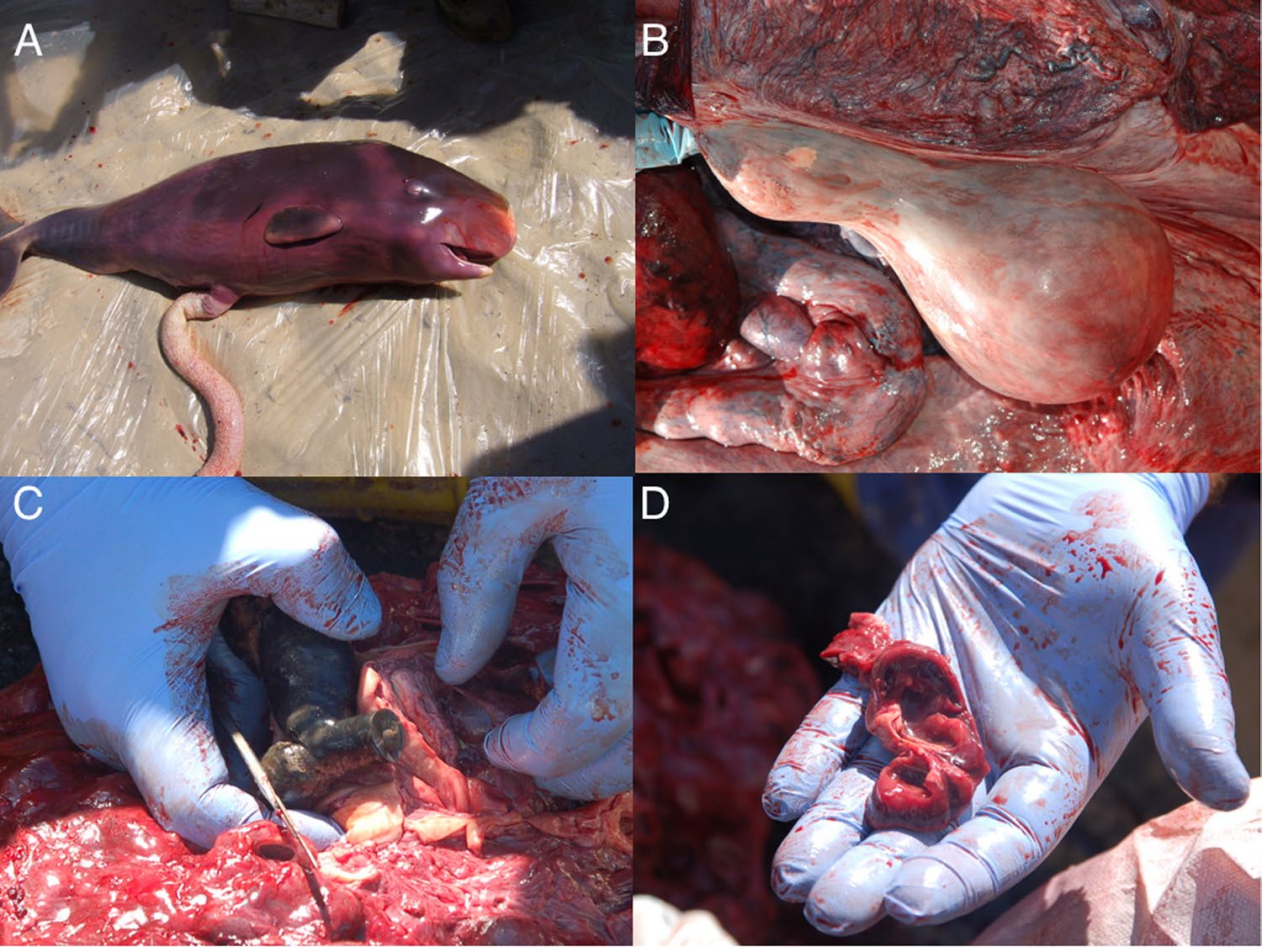
Necropsy findings. Some of the most relevant gross post-mortem findings are here shown: (**A**) the male fetus found in the pregnant SW1; (**B**) an ovarian cyst in the left ovary of SW2; (**C**) a close image of the renal stone found in SW1 along with (**D**) renal hydronephrosis affecting a single reniculus.

SW1, the oldest and pregnant whale (Fig. 1A), showed evident pathological changes. A greenish-black calculus, weighting 1,850 g, was found in the left renal pelvis of this animal, associated to compression of the parenchyma of the left kidney and marked and diffuse pelvic dilation of most of the renicula of the same side (Fig. 1C and D). Chemical analyses of the stone revealed an ammonium oxalate composition. Histopathology confirmed the diagnosis of hydronephrosis, accompanied by prominent fibro-sclerotic changes of the parenchyma, with mineralization of the cortical layer, and extensive loss of the medulla. Endothelial mineralization was additionally observed in vessels of the ovaries and lungs.

A luteinic cyst with a diameter of 8 cm was observed in the left ovary of SW2 (Fig. 1B).

A relevant megakaryocytic population was observed within the splenic parenchyma of SW3.

Systemic lymphadenopathy was noted in all the 3 whales, characterized by a diffuse greenish discoloration and the onset of a black pigmentation scattered on the cut surface. Histochemically, these dark pigments resulted strongly positive to Danscher’s staining method^21^, thus suggesting the presence of inorganic mercury. Similar pigments were observed also in samples of cerebral tissue collected from SW1 and SW2: brown droplets filled the cytoplasm of neurons. Periodic-Acid Schiff (PAS), Schmorl’s and Danscher’s stains revealed the dual/associated occurrence of inorganic mercury and lipofuscins. A relevant and diffuse lymphocytic depletion, accompanied by histiocytic infiltration of the sinuses, was also observed in lymph nodes of all whales. Clumps of eosinophils were detected in superficial cervical and mesenteric lymph nodes. Microscopic analyses of cerebral tissue and samples from other organs revealed the existence of mild and non-specific degenerative changes, coupled with rare and/or occasional lymphocytic infiltrations around brain vessels.

**Figure 2:**
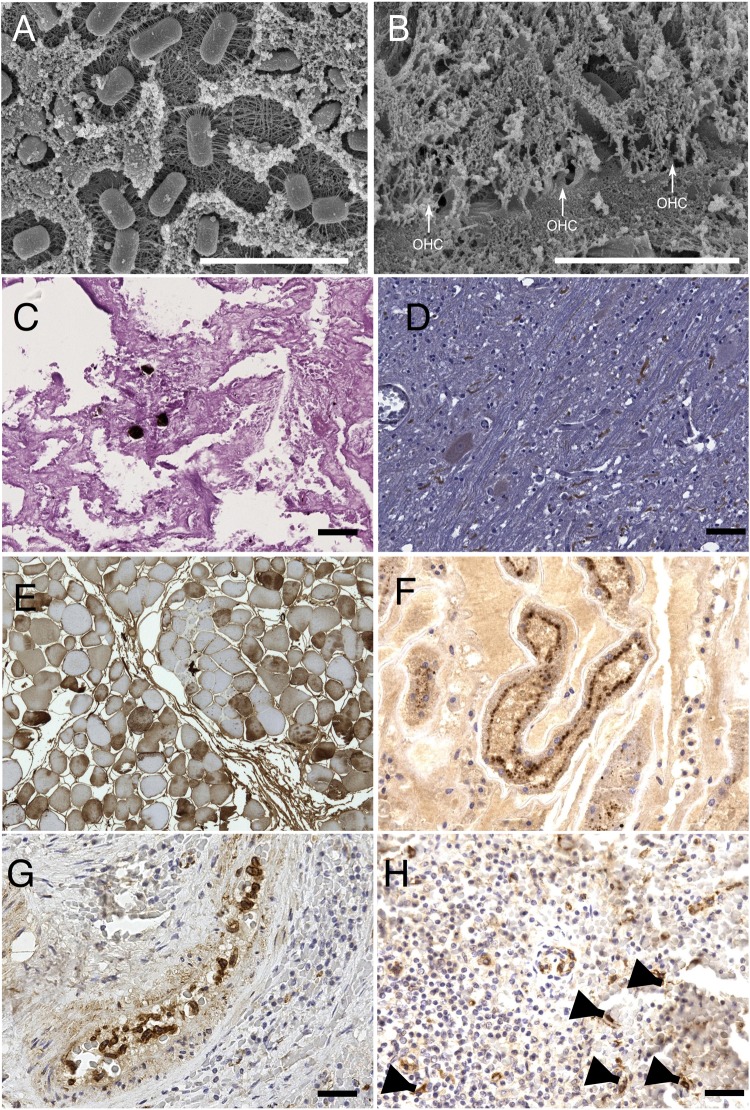
Microscopic findings. Some of the most relevant microscopic findings revealed by scansion electron microscopy (SEM), histopathology, and immunohistochemistry (IHC) examinations: (**A**) evidences of post-mortem bacteria proliferation and (**B**) post-mortem degeneration of Organo del Corti outer hear cells (OHC, white arrows) observed at SEM. (**C**) occasional fat emboli within pulmonary capillary vessels of SW3 revealed by OsO4 post-fixation technique, PAS 20×. (**D**) Positive immunostaining of axons using anti-caspases-3 antibody, suggesting ongoing apoptotic changes in in SW2’s brain; 40×. (**E**) IHC on muscular tissues revealed a multifocal cytoplasmic immunostaining within fibers by using anti-fibrinogen antibody suggesting an ongoing damage (40×); (**F**) rabdomyolisis was further confirm by IHC on kidneys using anti-myoglobin antibody: the picture shows myoglobin granules in the apical side of tubular epithelial cells (40×); (**G**) positive IHC staining of circulating monocytes in the spleen of SW3 by using anti-CDV antibody (40×); (**H**) this image shows the same results of the previous image in dendritic cells of SW3’s spleen (arrows), 40×.

Rare fat emboli observed in the lung parenchyma from SW3 (Fig. 2C), along with bubbles observed during gross examination of subcutaneous, mesenteric and coronary vessels, were possibly caused by the stranding. Tissue distribution and count of the bubbles are summarized in Table 4. Further laboratory analyses on sampled gas collections could not be performed due to the poor preservation conditions of the tissue samples under investigation.

**Table 4:** Gas bubble score: intra-vascular bubbles amount was estimated^45,46^ several venous vessels and a total score was finally calculated.

	Subcutaneous veins	Mesenteric veins	Coronaric veins	Lumbo-caudal venous plexus	Extra-vascular air	Total
SW1	3	2	2	1	2	10
SW2	4	2	3	0	1	8
SW3	3	2	3	0	1	7

Multi-level electron microscopy scanning (SEM) at of the cochlear spiral from SW2 revealed numerous extracellular bacilli overlying the apical aspect of the organ of Corti, the hearing organ (Fig. 2A). These bacteria could not be classified on a morphological basis. The examined sample showed no evidence of associated inflammatory response. Based on the degree of autolysis, the bacteria were deemed to be consistent with *post-mortem* overgrowth, rather than being considered as primary or secondary pathogens. The presence of any eventual *ante mortem* noise-induced hearing loss, wherever existent, could not be determined, since bacterial overgrowth obscured the organ of Corti, and decomposition had already set in among the few remaining hair cells of the small portion of the cochlear spiral (Fig. 2B).

Apoptotic changes were revealed in brains by the positive immunoreaction of axons to a commercially available anti-caspases-3 antibody (Fig. 2D). Rhabdomyolisis, along with renal endotubular myoglobin casts, myoglobinuria and muscular damages, were detected by immunohistochemistry (Fig. 2E,F), thus suggesting a stress-related, stranding-induced syndrome in all the examined whales. All these findings confirmed that all the animals stranded alive.

Finally, clear-cut evidence of morbilliviral infection was found in circulating monocytes (Fig. 2G), splenic macrophages, and dendritic-like cells from SW3 (Fig. 2H), following utilization of a commercially available monoclonal Ab against the N antigen of canine distemper virus (CDV), as reported elsewhere^22^.

### Virological and microbiological analyses

Biomolecular (RT-PCR) evidence of DMV infection was obtained from SW1–3 and the SW1b, as already reported elsewhere^23,24^. Molecular analyses carried out on SW1b showed evidence of herpes virus infection in the fetal liver and kidney with 100% homology of viral sequence with dolphin alpha-herpesvirus. (GenBank Acc No AY952779.1) No viral particles were observed with electron microscopy. Inoculated cell lines showed no cytopathic effect in three passages; cryolisated of cells lines were negative at molecular analysis for DMV.

Microbiological examinations did not reveal the presence of any other significant pathogens, with the only exceptions of *Photobacterium damselae* subsp. *damselae*, *Vibrio* spp. and *Aeromonas* spp., which were recovered from the undamaged tissues and organs of all the three sperm whales. Interestingly, *Clostridium perfrigens* and its exotoxins and other clostridial alfa-toxins were detected from the kidneys of SW1b (beta-toxin), SW2 and SW3 (alpha-toxin). *Brucella* spp. and *Salmonella* spp. were not isolated from any of the four individuals under investigation.

### Parasitological analyses

Specimens of *Neocyamus physeteris* (Crustacea, Amphipoda) were isolated from the skin of all the whales. Merocercoid larvae of *Phyllobothrium delphini* and *Monorygma grimaldii* (Cestoidea, Tetraphyllidea) were found in the blubber and mesentery of SW1 and SW2, respectively. Lesions caused by copepods belonging to the genus *Pennella* (Siphonostomatoidea, Pennellidae) were detected in the blubber of all the animals. A sample of 150 nematodes were recognized as *Anisakis* spp. specimens and they were subsequently identified by diagnostic allozymes as *A*. *physeteris*. Molecular analyses carried out on mtDNA *cox2* gene showed a 99% and 98% homology with two sequences previously deposited in Genbank^25,26^, confirming the results obtained with the allozymes. Pre-adults and adults of *Anisakis physeteris* (Nematoda, Anisakidae) were isolated from the stomach chambers of all the animals. Some *Anisakis* specimens were also detected, mostly spoiled and dead, among the cephalopod beaks collected as stomach contents. *Anisakis* eggs were observed in feces. All the 3 sperm whales resulted infected (Prevalence, P = 100%) by *A*. *physeteris*, and mean abundance values (A) of infection resulted, respectively, A = 740 in SW1, A = 650 in SW2, and A = 730 in SW3. Fecal smears from SW1 and SW3 were also positive to MZN, but the molecular analyses did not confirm the presence of *Cryptosporidium* DNA. Finally, the tissue samples examined for *Apicomplexa*, including *T*. *gondii*, resulted negative on PCR analysis.

### Gastric content analyses and biotoxin investigations

SW1–3 gastric contents resulted negative for all the tested biotoxins. In all the examined whales we digested food remains were found, along with a greenish-aqueous fluid. More in detail, squid beaks and lenses were found without any remain of soft tissues, thus suggesting no recent feeding activities (1858 g in SW1; 1465 g in SW2 and 403 g in SW3, respectively). Prey residues were more digested in SW2 than in SW1 and SW3. Total numbers of upper and lower cephalopod beaks were 3969 and 4469 respectively in SW1; 3599 and 4200 in SW2; and 1605 and 1528 in SW3. SW1 had also foreign bodies in its stomachs (1340 g of plastic bags, 1 jute bag and a piece of rope).

Only eight cephalopod species were present; of the 7,539 recognizable lower beaks, over 95% were classified in the family Histioteuthidae (71% to *Histioteuthis bonnellii* and 24% to *H*. *reversa*), followed by *Ancistrocheirus lesueurii* and *Octopoteuthis sicula*. Among these species, the largest one is *H*. *bonnellii*, which can attain a size of more than 6 kg in weight, while all the others are much smaller, rarely exceeding 1 kg in weight.

All these cephalopods are meso- or bentho- pelagic species, inhabiting deep waters and are not present in the Central and Northern part of the Adriatic; it is therefore evident that they were preyed upon in the Ionian Sea or in the Southern Adriatic.

### Ecotoxicological data

**Figure 3:**
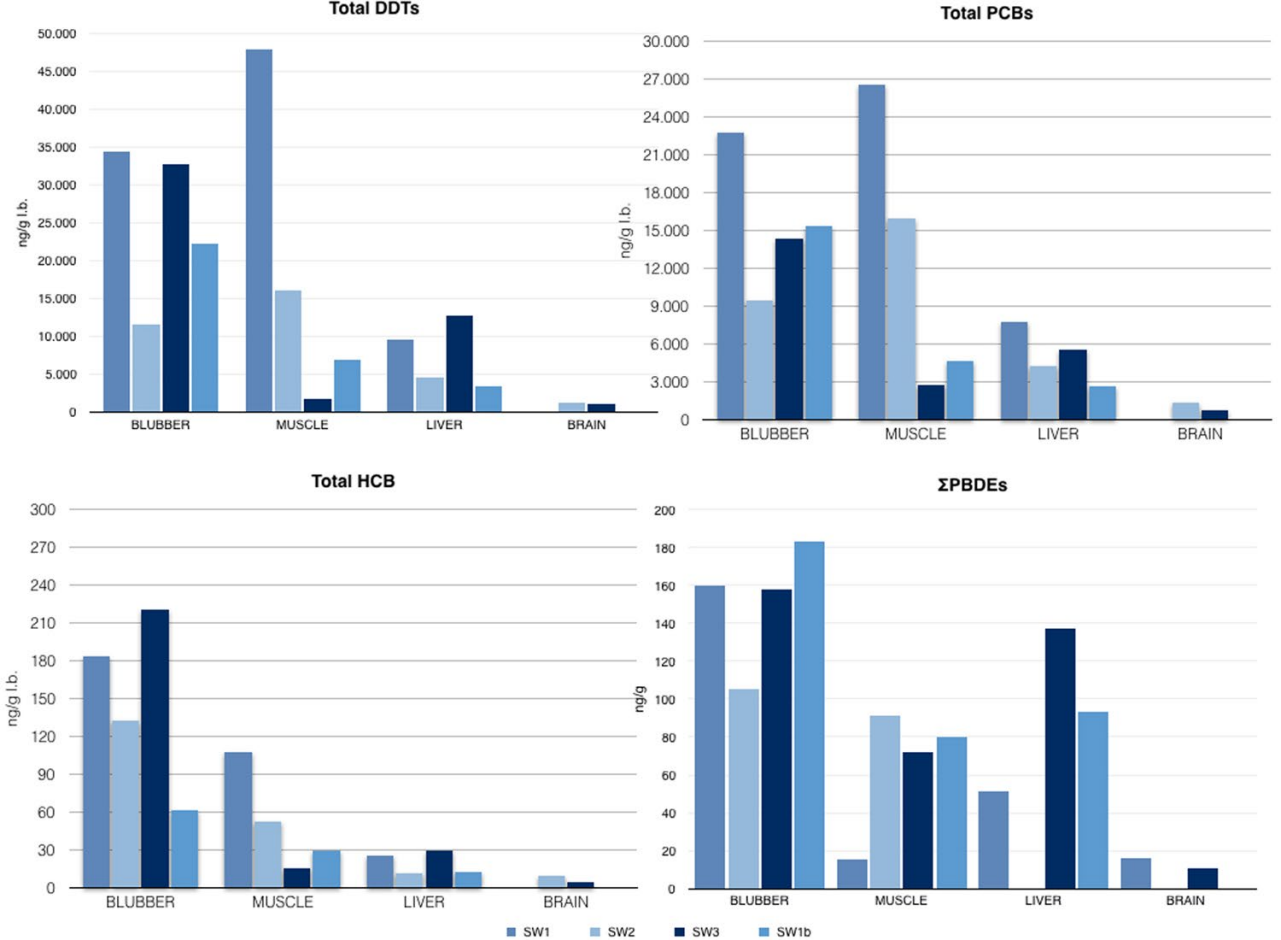
Ecotoxicological analyses. Graphics herein represented summarize respectively DDTs (upper left), PCBs (upper right), HCB (lower left) and PBDEs (lower right) concentrations in tissues of SW1, SW2, SW2 and SW1b. Data are summarized in Tables 1 and 2 in Supplementary Tables S2 and S3.

In the present paragraph results obtained by toxicological examinations are resumed and showed in graphics in Fig. 3. Data are also reported in Table 2 in supplementary materials.

#### Hexa-chloro-benzene (HCB), dichloro-diphenyl-trichloroethane compounds (DDT) and poly-chloro-biphenyl compounds (PCB)

The concentration of organochlorine compounds obtained analyzing subcutaneous, hepatic, muscular and renal tissues of SW1, SW2 and SW3, and brain samples of SW2 and SW3, are shown in Fig. 3 and Supplementary Table S2. DDT concentrations were higher than PCBs and HCB, except for muscle samples of SW3. The main component was pp’ DDE (81%, 84%, 78% and 83% of the total in SW1, SW1b, SW2 and SW3, respectively).

#### Polybrominated diphenyl ethers (PBDEs)

The presence of flame retardants was evaluated in blubber, muscle and heart of SW1–3 and SW1b, and in the liver of SW1, SW2 and SW3 (Fig. 3). The highest total amount was observed in the blubber of all individuals, including the fetus, especially in SW3 (Supplementary Table S3). Most represented PBDE classes were 47, 99, 100 and 154.

#### Dioxins (PCDD e PCDF)

Table 5 summarizes the main parameters used for evaluation of a) the dioxin burden (PCDD and PCDF and dioxin-like PCBs), as suggested by the World Health Organization; and b) the non-dioxin-like PCBs, considered as the sum of the six PCB indicators usually determined for the evaluation of PCB contamination.

**Table 5:** Toxicity Equivalent Quantity (WHO-TEQ-05) of Dioxins (PCDD, PCDF, DL-PCB*), and SUM ICES-6 of non dioxin-like PCBs (NDL-PCB**) in whole weight of blubber and spermaceti of sampled Sperm whales.

Parameter	SW1-Blubber	SW2-Blubber	SW3-Blubber	SW3-Spermaceti
Dioxins + dioxin-like PCBs TEQ (pg/g ww)	118,78 ± 15,51	88,92 ± 12,45	185,09 ± 24,42	340,57 ± 44,93
Dioxins TEQ (pg/g ww)	26,35 ± 4,40	19,59 ± 3,27	38,68 ± 6,46	71,21 ± 11,89
Dioxin-like PCBs TEQ (pg/g ww)	92,43 ± 14,87	69,33 ± 11,15	146,41 ± 23,55	269,36 ± 43,33
Non dioxin-like PCB indicators (ICES-6, ng/g ww)	807,8 ± 110,2	911,0 ± 131,0	1067,9 ± 138,2	2404,9 ± 314,3

*PCDD: 2,3,7,8-TCDD; 1,2,3,7,8-PeCDD; 1,2,3,4,7,8-HxCDD; 1,2,3,6,7,8-HxCDD; 1,2,3,7,8,9-HxCDD; 1,2,3,4,6,7,8-HpCDD; OCDD). PCDF: 2,3,7,8-TCDF; 1,2,3,7,8-PeCDF; 2,3,4,7,8-PeCDF; 1,2,3,4,7,8-HxCDF; 1,2,3,6,7,8-HxCDF; 1,2,3,7,8,9-HxCDF; 2,3,4,6,7,8-HxCDF; 1,2,3,4,6,7,8-HpCDF; 1,2,3,4,7,8,9-HpCDF; OCDF). Dioxin-like PCB: PCB-77; PCB-81; PCB-126; PCB-169; PCB-105; PCB-114; PCB-118; PCB-123; PCB-156; PCB-157; PCB-167; PCB-189.

**Non dioxin-like PCBs (ICES-6): PCB-28; PCB-52; PCB-101; PCB-138; PCB-153; PCB-180.

On average, the dioxin burden of specimens was mainly due to the dioxin-like PCBs (78.3%; min-max values: 77.8–79.1%) while PCDD and PCDF contributed for 21.7% (min-max values: 20.9–22.2%), independently of the matrix. Spermaceti revealed greater dioxins concentrations than the bubbler.

#### Algal biotoxins

Analyses carried out on stomach contents of SW1–3 revealed neither harmful algal bloom (HAB)-related/associated hydrophilic nor lipohylic biotoxins.

## Discussion

The sperm whale mass stranding described in the present report is only the 7^th^ that was recorded along the Adriatic coastline since 1555^14^. Interestingly, another such event occurred as recently as 2009 along the Southern Adriatic coastline^12^. The causes of mass strandings are difficult to understand: viral infections and chemical pollution have been already considered to play a crucial role^24,27^. However, a complete investigation of the underlying causes requires a thorough multidisciplinary, multi-analytical effort to draw solid and reliable hypotheses^15^.

Seven animals were involved both in the 2009 and in the 2014 strandings. However, the social structure of the two stranded pods, inferred by biological and genetic information, was different. The 7 whales stranded in 2009 were all juvenile males forming a “bachelor’s school”, while in the most recent event the pod was composed by at least 3 females, with an older one, (SW1, most likely the leader), one subadult (SW2) and one juvenile (SW3); the 4 refloated specimens could have been all juveniles, possibly both males and females, representing the typical composition of a “breeding school”, as defined by Whitehead and coworkers^28^. This latter hypothesis, based on biological data, was supported also by genetic and stable isotope analyses. More in detail, sperm whales have a very low variability of the mitochondrial DNA control region compared to other cetacean species. Several studies detected no variability in the Mediterranean sperm whale population^16,29,30^. This means that mtDNA was not able to provide unequivocal information about matrilineal relatedness within our group of study. For these reasons, a parentage analysis was attempted, and the results suggested that the stranded whales likely belonged to a family pod, based on fullsib relationship between SW2 and SW3 and the kinship with SW1. Remarkably, SW1, SW2 and SW3 shared the same father. No relationship was noted with the pod stranded in 2009. However, here we note that 1 individual involved in the 2009 stranding shared the same father with a female stranded in 2008 along the Tuscany coastline.

Stable isotope analyses also support the Mediterranean origin of the stranded pod: the skin δ^13^C values determined in this study were similar to those reported by Pinzone and colleagues^31^ for sperm whales collected in the North Western Mediterranean Sea, whereas less negative δ^13^C values were observed by Ruiz-Cooley and colleagues^32^ for sperm whales stranded in the Gulf of California, due to a different diet. Sperm whales are teuthophageous, and in the Mediterranean feed preferentially on meso- and bathy- pelagic cephalopods of the family Histioteuthidae^33^. δ^13^C isotopic variations reflect differences of food sources and habitat distribution (nearshore vs. offshore). Nevertheless, a depletion of ^13^C and ^15^N may occur in tissues under fasting- and nutritional-induced stress, when adipose stores are used as energy source. Under these conditions, an increase of ^15^N may take place when the catabolism of protein prevails through the gluconeogenesis pathway^12,28^.

The above reported observations could be related either to SW1 pregnancy, or to the pathological changes observed at *post-mortem* examination. This female, in fact, showed a severe monolateral hydronephrosis, with concurrent fibrotic changes of the kidney parenchyma, caused by a large renal stone. Cetacean kidneys are essential not only for the osmoregulation, but also for hydration. Nevertheless, urinary tract lesions are infrequently reported in these mammals and, whenever diagnosed, they are often associated to systemic conditions^34^. In SW1, the features and the extension of the renal damage, associated with pregnancy and a reduced food intake (the latter meaning dehydration in cetaceans), could have led to a severe renal function impairment. The pathophysiology of the renal calculus (i.e. ammonium oxalate) is largely unknown in marine mammals. Human uric acid calculi originate from oxalate concretions and account for a significant percentage of urinary stones with hyperuricosuria, low urinary volume and persistently low urinary pH as risk conditions. Etiological factors include genetic disorders, chronic diarrhea, dietary excess, insulin resistance, primary gout and metabolic disorders of uric acid. Possible symptoms and signs associated to this urinary stone include pain, malaise, nausea, lower urinary tract symptoms, and hematuria^35^.

Beside this potentially invalidating condition, SW1 and all the examined whales, including the foetus, were affected by DMV infection^23,24^. The pathological changes typically associated with such infection were not observed in the stranded whales. However, a viremic condition was strongly suspected based on biomolecular and microscopic investigations. More in detail, the hypothesis of a DMV- related viremia was supported by a) positive immunolabeling of morbilliviral antigen in intravascular monocytes as well as in splenic macrophages and follicular-like dendritic cells; and b) by the absence of positive IHC staining in epithelial and nervous tissues. The viremia was likely characterized by an active viral replication in immune cells, being presumably associated to a condition of pronounced distress in SW1, SW2 and SW3^36,37^. The concurrent finding of a herpesviral infection in the foetal individual indirectly confirms the poor health status of the oldest pregnant female, since herpesviruese are frequently detected in immunocompromised hosts and often reported in association with DMV infection^38,39^.

The DMV infection reported in all the examined animals was probably an important co-factor in the mass stranding^24^ as well as in the debilitating condition affecting SW1. The different isotopic profiles reported for SW1 and the other 2 whales do not support a severe and prolonged fasting. Analyses of gastric contents with the consistent presence of pre-adults and adults of *Anisakis physeteris* nematodes in the stomachs of all the whales indicate that the whales fed in the recent past, but not in the days immediately preceding the stranding. Cephalopod beaks observed in their stomach belong to species living in the Southern Adriatic Sea: if the stranded pod is the same reported along the Croatian waters in the Central Adriatic basin on September 9^th^, a fasting period of at least 7 days could be hypothesized.

These observations also confirm the specificity of this parasite species for this mammalian host and reveal how *A*. *physeteris* life cycle is embedded with the ecological characteristics of sperm whales, having squid rather than fish as suitable intermediate hosts^40^. The presence of *Cryptosporidium* oocysts on fecal smears was not confirmed by molecular methods. Therefore, further analyses would be recommended to precisely identify the nature of the observed parasites. Pinnipeds and sirenians are the marine mammals most exposed to the risk of *Cryptosporidium* infection, since they spend time on the mainland or in coastal waters. However, this parasite was found also in cetaceans, i.e. bowhead whales, North Atlantic right whales, minke whales, bottlenose dolphins and striped dolphins^41,42^. Here we emphasize that *Cryptosporidium* oocysts have never been observed in sperm whales^43^.

Interactions with anthropic sound sources (i.e. military sonars and airguns used during seismic surveys) were excluded based on the absence of related pathological changes at *post-mortem*, and on official communications from Governmental Institutions of Adriatic riparian States. More precisely, the “gas and fat embolic syndrome” was adequately ruled out based on the distribution and quantification of gas bubbles as well as of fat emboli in the circulatory system^44^. Lipid droplets were indeed found in pulmonary capillaries in SW3, but their amount and distribution rather suggest a damage related to the prolonged recumbency. The rolling movements of the stranded cetaceans due to the marine currents and waves injured the corresponding/ipsilateral adipose and soft tissues around the jaw, likely determining a subsequent lipid embolism. The distribution and quantification of the bubbles observed in the vessels are indicative of a *peri-mortem* change. No chemical analyses could be performed on the air samples collected during necropsies due to field conditions, but the evaluation of gas bubbles in the vessels according to Bernaldo de Quiros and colleagues^45,46^ excluded air embolism due to rapid ascent. Furthermore, researches and queries to Governmental Authorities, carried out in the two years after the event, indicate that no activity involving underwater sound sources was ongoing at the time of stranding, thus ruling out acoustic traumas and subsequent disorientation as causes of the stranding.

A previously published paper regarding this mass stranding^27^ considered that methylmercury (MeHg) and other heavy metals could have been possible relevant co-factors. The Hg tissue concentrations measured in SW1, SW2 and SW3 are relatively high compared to those of terrestrial mammals. However, hepatic and renal total Hg and MeHg values are similar to those previously reported in Mediterranean sperm whales^12,31,47,48^, albeit higher than those measured in sperm whales involved in mass strandings that took place in the North Sea^49^. Even if MeHg may have reached the tissues of SW3 through blood or lymph, its relative percentages on total Hg, especially in the kidneys, are lower than those reported in the mass stranding occurred in 2009^12^. In that situation, the body circulation of MeHg originating from diet and body storages was associated with a lower renal MeHg excretion caused by a lower food (and, consequently, water) intake, with subsequent modulation of glutathione complex and thiol activities, due to prolonged fasting^12^. Squadrone *et al*.^27,50^ reported that MeHg percentages in SW1–2 were lower than those reported in the liver and kidneys of the whales stranded in 2009^12^. The only exception is SW3 (which also had less gastric residues), showing MeHg concentrations comparable to those of the 3 whales examined in 2009 (respectively PM5: 15 yo; PM6: 20 yo and PM7: 20 yo).

As stated above, the hypothesis of a prolonged fasting for the entire group was not supported by stable isotope and gastric contents analyses, as well as by the parasitic burdens in the gastric chambers. For these reasons, the higher amount of circulating MeHg in SW3 could be related to individual features like age, food intake and/or pathological conditions, but cannot be considered as a relevant cause of the mass stranding.

Interestingly, the circulation of inorganic mercury, suggested by microscopic evidences of cytoplasmic accumulation of black pigments positive to Danscher’s staining in lymph node macrophages, hepatic Kupffer cells and neurons, was theorized in the 2009 stranding, and may apply also to the herein reported event. Since no hepatic degenerative changes were observed and fasting was not hypothesized, these findings support the theory that an increase of the inorganic Hg fraction in lymph nodes is likely due to the demethylation process of MeHg taking place in macrophage-rich lymphoid tissues^51^. Our present data suggest that exposure to MeHg prompts a preferential accumulation of inorganic Hg within lymphoid tissues in comparison to other non-lymphoid body districts of the whales^52^.

Nevertheless, we cannot definitively rule out that MeHg, a recognized immunotoxic pollutant, was responsible for the widespread lymphoid cell depletion observed in SW1–3. The same may apply also to the high concentrations of the organic pollutants (DDTs, PCBs, dioxins, and flame retardant) found in tissues, even if OC levels were lower than those reported in the previous sperm whale stranding of 2009^12,53^. In particular, PCB and DDT levels in the blubber were lower in the females stranded in 2014 than in the males stranded in 2009 (respectively an average value of 25 ppm vs 205 ppm for DDTs and 16 ppm vs 194 ppm for PCBs), even if OC contents varied greatly among the individuals. In both stranding events, DDTs were more represented than PCBs, supporting the hypothesis of a more effective excretion of the latter ones but also of a preferential accumulation of DDTs through diet. Sperm whales feed on a narrow range of prey, but diet composition may differ with location and season, and bulls may feed on larger preys^26^. Squids are preferred preys in the Mediterranean Sea. Since the DDT levels in these cephalopods are normally higher than those of PCBs, the input of this xenobiotic through the food route is more relevant^54^. A very interesting result, emerging from the observation of DDT metabolites, is the relationship between pp’DDE and pp’DDT. A typical technical DDT is composed of pp’DDT (77.1%), op’DDT (14.9%), pp′DDD (0.3%), op’DDD (0.1%), pp’DDE (4.0%), op’DDE (0.1%) and unidentified compounds (3.5%) (WHO, 1979) and the pp’DDE/pp’DDT ratio is 0.05. A high pp’DDE/pp’DDT ratio states that most of the active substance (pp’DDT) has been degraded to pp’DDE, and therefore that no insecticide recently entered the ecosystem^55^. In all the examined specimens, pp’DDE/pp’DDT ratio resulted always higher than 10, reaching the value of 46 in the liver of SW2, and this ratio indicates an old DDT contamination. The pp’DDE/DDTs ratio, differently from the pp’DDE/pp’DDT ratio, can also indicate the efficiency of the metabolic processes of a population^56^. In the stranded whales the pp’DDE/DDTs ratio varied from 0.78 to 0.86, confirming a very high metabolism of this pesticide. The PCB and DDT burdens measured in our samples were higher than those documented in this species in previous non-Mediterranean studies^9,49,57^ but lower than those found in the sperm whales stranded along the coast of Apulia in 2009^53^. However, differences in analytical techniques, or in the PCB congeners evaluated and/or DDT metabolites and tissue analyzed make any comparison challenging. It is also true that the sperm whales from the 2009 mass stranding were all males, while the specimens of this study were all females and a foetus. The fact that CH levels are normally higher in males than females is explained by the fact that females lose up to 90% of their total body burden of these substances during pregnancy and lactation, while males accumulate them throughout their lives^58,59^. A statistically significant difference of age and sex-related bubbler OC loads was also recently reported by Pinzone and colleagues^31^ in free-ranging Mediterranean sperm whales. The oldest female (SW1) showed the highest PCB and DDT levels among the whales stranded in Vasto, besides the OC content of her foetus. These results document a high level of these compounds in SW1 sperm whale, that match the highest values recorded in Mediterranean females of the same species^31^. The concentrations of PBDEs were also higher in the adult pregnant female than in the two younger individuals. Data on PBDEs in sperm whales are rarely reported, but our results indicate a relevant diaplacental transfer, as described in beluga whales (*Delphinapterus leucas*)^60^. Pathological changes observed in the ovary of SW2 further support the role of PBDEs as endocrine disruptors^61^.

No differences were noted for HCBs, thus confirming the low concentrations already reported in other large Mediterranean marine mammals^12,53,62,63^.

Dioxins and dioxins-like compounds are considered a possible threat for terrestrial environments during local environmental disasters (burning of wastes, illegal industrial releases, etc.). Some studies on Mediterranean fish^64^ and mammals^31^ showed that the marine environment is not exempt from dioxin pollution. Even if their effects on the stranded whales could not be evaluated, data on dioxin loads in bubbler reveal a greater proportion of PCDD/F on total TEQ (21.7%) compared to the reference study by Pinzone *et al*.^32^ on individuals of the same species (13%) as well as to data obtained from bottlenose dolphins stranded in the Adriatic Sea in 2014 (1.3%).

In conclusion, our data support the hypothesis of a “follow me”-related mass stranding, meaning that the pod followed a sick individual, likely the leader of this familiar group, towards an acoustically dead shore after spending debilitating days in the less-than-optimal Central Adriatic Sea, This “sick leader syndrome”-related hypothesis is supported by the life-threatening findings noted in the oldest female and by the good health conditions observed in the other individuals, documented by the results of the *post-mortem* examinations and by the success of the re-flotation attempts on the remaining 4 whales. SW1 showed relevant pathological changes in its urinary tract, which – in combination with the heavy metabolic demand of pregnancy and the dehydration related to fasting - likely led to a severe renal functional impairment in this animal. The tissue concentrations of MeHg and of the investigated OCs may have been not relevant enough to affect the health and behavior of SW1 and of the entire group. On the other hand, the DMV infection detected in all the examined whales likely played a pivotal role in changing the orientation capabilities of SW1, thus worsening its already poor health condition and favoring the entrance of the entire pod into the Adriatic Sea, with subsequent mass stranding along the Central Adriatic coastline.

Finally, the hypothesis herein proposed underlines the importance of evaluating and discussing data using standardized protocols, as in the event described here. Evidences should result from thorough and detailed *post-mortem* and laboratory investigations, coupled with a correct and unbiased collection of data and information, to be interpreted using a multidisciplinary approach^15^.

## Methodology

Detailed *post-mortem* investigations were carried out on the 3 sperm whales that died on the stranding (SW1, SW2 and SW3). Other 4 individuals (SW4–7), belonging to the same pod, were successfully refloated after great efforts. During necropsies, a *fetus* was found in SW1 and it was labeled as SW1b.

Necropsy procedures and related analyses followed an already established protocol defined during a previous mass stranding of the same species^12^. Furthermore, detailed and exhaustive investigations were performed based on the previous experience to reach a thorough understanding of the event.

Tissues for the following analyses have been preserved and provided by the Mediterranean Marine Mammal Tissue Bank (CITES authorization no. IT020), Department of Comparative Biomedicine and Food Science, University of Padova, viale dell’Università 16, 35020 Legnaro - Agripolis PD, Italy.

### Logistic organization on the field

Re-floatation was achieved after evaluation of the vitality and size of each whale. Volunteers on the field dug furrows around the whales to allow careful swaying towards shallow waters. Coast guard vessels subsequently gently pulled the animals towards deeper waters (up to a depth of approx. 5 m) thanks to specific lifting bands passed around the head, behind the eyes, and across the abdomen. Other volunteers entered the waters and monitored the initial progress of each whale into the sea to remove any possible obstacle. The sperm whales were then freed from all harnesses and released in high waters, under kept continuous surveillance from a group of zodiacs. Another team maintained communication between operators on the shores and boats, thus ensuring overall coordination of all operations.

*Post-mortem* examinations were conducted on the beach after consultation with the local Authorities and considering disposal options for the carcasses according to EU Directive 1069/09. A total number of 65 operators participated in the field operations, including veterinarians, biologists and student volunteers. The Cetacean stranding Emergency Response Team (CERT), established at the University of Padua in 2011 from the Italian Ministry for the Environment, was appointed as On-Scene Coordination Body (OSCB) according to ACCOBAMS resolution 4.16. Follow-up analyses, described in the specific sub-chapters below reported, involved 18 different Institutions.

During *post-mortem* operations, persons on the field were divided in different teams, each coordinated by an appointed team leader. The teams consisted of: *a*) 3 different necropsy teams of 4 persons each performed gross examination of the bodies - except the head - and collected photographic and written documentation; *b*) a specific team of 3 persons opened the heads and extracted the inner ears; *c*) a sampling groups of 10 persons was dedicated to receiving, labelling and processing samples for all ancillary analyses detailed in the following paragraphs; *d*) a logistic team composed of 2 persons took care of all disposable materials and sharpening of knives; *e*) a connection team of 9 persons ensured transportation of the samples and provided maintenance of the equipment; *f*) a team of 2 photographers documented the events; and, finally, *g*) 1 person took care of the communication between the OSCB and the different teams and 2 persons were selected to provide food and beverages. Furthermore, local NGOs informed the press and the general public on the nature and progress of the operations.

Cranes were used to move the bodies of the dead sperm whales to an area then limited to necropsy procedures. The setting included also *a*) a logistic point where all equipment was available; *b*) a sampling point with a wet part and a clean area; *c*) a shadowed area dedicated to resting and storage of personal belongings.

### Post-mortem protocols

In-depth necropsies were carried out on the 3 dead sperm whales following standard protocols adapted to sperm whales^12,16^. The heads were separated from the trunk and the brains were then removed cutting the occipital condyles with a chainsaw^65^. A comprehensive sampling of all organs and tissues was performed for later microscopic examinations. Given the importance of the inner ear, a special group was dedicated to remove the inner ear of SW2. Histopathological, immunohistochemical and ultrastructural studies were performed on tissues fixed by immersion in aldehydes. The eventual presence of *Morbillivirus*, *Herpesvirus* and *Toxoplasma gondii* was investigated by virological, microbiological and biomolecular techniques. Specific attention was payed to the presence of gas emboli by gross examination of subcutaneous, coronaric, mesenteric, renal and iliac veins, as well as of any gas collection in other organs. Gas bubbles were sampled from the coronary veins by vacutainer H tubes^66^ and findings were quantified and described according to de Bernaldo de Quiros and colleagues^44,45^. Lung tissue samples fixed in formalin were used to investigate the presence of lipid emboli. Furthermore, sampling for age determination as well as genetic, stable isotopes, diet and toxicological analyses were collected fresh or frozen. A complete list of all the samples collected during necropsies, their preservation and the subsequent exams carried out are included in supplementary material (Table 1).

### Age and weight estimation

All the dead animals were measured before *post-mortem* examinations and the total lengths of the 4 surviving whales SW4, SW5, SW6 and SW7 were estimated by skilled and trained volunteers before refloatation attempts. The total length was used to estimate the weight of each whale according to a formula specific for the species^67^. Furthermore, the carcasses and the remains of SW1, SW2 and SW3 were individually weighted during their disposal to obtain a “corrected” weight inclusive of the fluid and tissue losses that inevitably occurred during necropsy procedures and transportation.

Two mandibular teeth collected from each sperm whale were sectioned along the sagittal plane, following two different methods. The first tooth was sectioned into two halves; one half was observed directly, and the other half was polished and then etched in 15% formic acid until clear, easily discernible dentinal layers or growth layer groups (GLGs) were obtained. The second tooth was mounted in epoxy resin and cut in several sections 0.5 mm thick, using an ISOMET Low Speed Saw endowed with a diamond blade. Sections were later scanned with a high-resolution scanner and digitalized.

The total number of GLGs in each of the tooth sections was determined in three separate sessions by three independent readers.

### Photo-identification

Several photos of the flukes of each whale were taken to record any available characteristic that could result in individual identification, such as marks along the trailing edge (nicks, notches, scallops, irregularities, etc.), and/or pigmentation patterns on both surfaces. Additional photos of the dorsal fin and lower jaws were taken to aid the identification process. The best photo of each stranded whale was compared to the photos available in three photographic databases of free-ranging sperm whales from the Mediterranean Sea for the years 1990–2009 (GREPHYSC 2009, Greek *Physeter* Catalogue; the Mediterranean part of NAMSC 2004, North Atlantic & Mediterranean Sperm Whale Catalogue; the Tethys sperm whale catalogue 2008, TeSC). A non-automatic comparison was performed by visual inspection of each photo, since the total number (a few hundreds) of known individuals in the Mediterranean Sea is relatively small.

### Genetics

Skin samples from SW1, SW2 and SW3 were analyzed along with other 4 samples from other individuals provided by the MMMTB of the University of Padua, as specified here below:

1) SWA: adult female, Piombino (LI), 2008

2) SWB: 24 years old male, Cagnano Varano (FG), 2009^12^

3) SWC: 19 years old, male, Cagnano Varano (FG), 2009^12^

4) SWD: 19 years old, male, Cagnano Varano (FG), 2009^12^

We performed the direct sequencing of a 399 bp portion of the mtDNA control region (Dloop)^68,69^. The sequences obtained were aligned with sequences previously obtained from Mediterranean sperm whales and deposited in the international GeneBank database.

Parentage analyses were performed on 16 microsatellite loci (EV3; FCB17; EV21, EV37, EV94, EV104; MK6; EV5; Sw19; FCB1; EV1; SW10; GATA417; GATA028; FCB14; TexVet5). These short tandem repeats (STR) have been characterized in different cetacean species^70–75^ and resulted polymorphic in preliminary analyses carried out on samples from SW1–3 using the software CERVUS v. 3.0.7^76^.

An exploratory analysis was also conducted by Factorial Correspondence Analysis (FCA) implemented in GENETIX 4.052.2^77^, to show eventual systematic relationships between the data obtained without entering *a priori* information about the nature of the data and possible relationships among them.

Reference database or a group of candidate parents were not available, hence, we used the sibship reconstruction approach implemented in the COLONY software, version 2.0.5.9^78^, to describe the relationship among the animals. The analyses were led using the Full likelihood method and simulations were performed with variable numbers of seeds, length and numbers of runs.

The age of the animals was used to provide paternity and maternity exclusion data. The analyses were then also performed without providing this information. The results relating to the best cluster, full sib and half sib were considered.

Finally, the Bayesian clustering algorithm implemented in 2.3.4 STRUCTURE^79^ was employed to assess the number of genetic groups in the sample. The posterior probability of the number of populations which best suits our genetic data (K) was calculated using the admixture model, which provides the opportunity for individuals to have common ancestors. The analysis was performed both by providing information on the location of sampling and without providing this information. Both the correlated allele frequencies model and the independent allele frequencies model were run^80^.

All runs were carried out by testing K (number of populations) from 1 to 5, with 5 × 10^5^ step of burn-in and 10^5^ MCMC iterations, with 5 replicas for each K investigated. The optimal value of K was estimated based on the method described by Evanno and colleagues^17^.

The F*st* differentiation factor between populations was calculated using the FSTAT Software version 2.9.3.2^19^.

### Isotope analyses

Lipids from blubber samples (0.02 mg) were extracted before analyzing carbon isotopes (δ^13^C), according to the Folch method modified by Boscolo *et al*.^81^, since lipids are depleted in ^13^C compared to the protein and carbohydrate fractions, thus giving rise to biased δ^13^C results. The isotope analyses were performed on bulk tissues and after extraction of lipids, to evaluate the data correction. The δ^13^C and δ^15^N signatures were determined at the ISPRA Laboratories of Chioggia with an Isotope Ratio Mass Spectrometer using a Delta V Advantage (Thermo Fisher Scientific, Bremen, Germany) interfaced with a Flash 2000 and Conflo IV (Thermo Fisher Scientific, Bremen, Germany). The analytical precision of measurements was 0.2%. Sucrose (IAEA-CH6, HYPERLINK “http://www.iaea.org/” International Atomic Energy Agency, Austria) and L-glutamic acid (RM 8574, National Institute of Standards and Technology NIST, Maryland, USA) were used as certified reference materials. The ratio of stable isotopes was expressed in delta notation (*δ* = [(*R*_sample_/*R*_standard_) − 1)] × 10^3^), where δ is the isotope ratio of the sample relative to the standards (international standard Vienna Pee Dee Belemnite for carbon and atmospheric nitrogen for nitrogen). R_sample_ and R_standard_ are the fractions of heavy to light isotopes in the sample and standard, respectively. One is subtracted from the R_sample_/R_standard_ fraction so that samples with a ratio of heavy isotopes lower than the standard have a negative value and those with ratios of heavy isotopes higher than the standard have a positive value. This number is then multiplied by 1000 so that the δ notation is expressed in units of parts per thousand (‰).

### Microscopic analyses (histopathology, histochemistry, immunohistochemistry and ultrastructural investigations)

Tissues samples were collected from a) body organs and b) macroscopically evident lesion sites of the 3 whales which underwent a full necropsy. Specimens were formalin-fixed, paraffin-embedded, cut into 5 µm-thick sections and then stained with haematoxylin and eosin for routine light microscopic evaluation. Periodic acid Schiff (PAS), Gram and Giemsa stains were also performed, to assess the presence of non-viral pathogens within the tissues. The presence of inorganic mercury deposits within tissues and cells was investigated by auto-metallographic techniques, including Danscher’s staining^20^ and X-ray microanalysis, performed using an Environmental Scanning Electron Microscope (ESEM) equipped with a fluorescence X-ray scanning system. Schmorl’s stain was used to evaluate the presence of lipofuscin pigments. Finally, lung samples stored in buffered formalin were post-fixed with osmium tetroxide (OsO_4_) to detect fat emboli^82^.

Selected tissue sections obtained from a) lungs and lymph nodes of all the 3 animals, b) the brains of SW2 and SW3 and c) the spleen of SW1, were additionally submitted to a detailed search for *Morbillivirus* nucleoprotein (N) antigen by means of a suitable immunohistochemical technique (IHC)^21^. IHC analyses were performed to detect any stress-related changes linked/associated to stranding, and to confirm that the animals stranded alive. Two different antibodies (Abs) were used for this purpose on skeletal and cardiac muscles, namely a polyclonal rabbit Ab raised against human fibrinogen (Dako, Agilent Technologies), which was used at a dilution of 1:200 and incubated for 2 hours at 37 °C, and a polyclonal rabbit Ab against human myoglobin (Dako, Agilent Technologies) that was employed at a dilution of 1:800 and incubated for 16 minutes at 42 °C. The anti-human myoglobin Ab was used also on sections of the kidneys of SW1–3, to detect the occurrence, if any, of intra-renal myoglobin.

### Virological and microbiological analyses

Methodology to reveal molecular evidences of DMV infection have been already reported elsewhere^22,23^. Further analyses were conducted to determine the eventual presence of any additional viral and/or bacterial pathogen that may have influenced the mass stranding. Investigations for *Herpesvirus* were performed using a consensus PCR method which amplifies a region of herpesviral DNA-directed DNA polymerase^83^. For electron microscopy, tissue samples were prepared following routine procedure^84^. Moreover, multiple organs were homogenated and inoculated on marmoset lymphocyte cell line (B95a) for viral isolation; after three passages the cryolisate of the cells lines were submitted for molecular analysis.

Investigations for standard microbiology and *Mycoplasma* spp. were carried out on specific tissues; furthermore, both viable and PCR products of *Brucella* spp. were carried out according to OIE Terrestrial Manual^85^.

### Gastric content analyses

The organic fraction of the stomachs contents was represented exclusively by cephalopod beaks. The beaks were washed, weighed and sorted according to three stages of digestion: complete, damaged and highly digested beaks. Only the first two stages were taken into account and divided into upper and lower beaks for counting. The lower beaks were identified to the lowest taxonomic level possible, according to Clarke^86^. The foreign bodies of anthropogenic origin were also weighed and sorted.

### Inner ear analysis

The right ear of SW2 was extracted and fixed in 10% neutral buffered formalin within 30 hours after death. The periotic bone surrounding the cochlea was decalcified for 9 days and 17.5 hours by using different dilutions of a specific rapid decalcifying agent RDO® (Apex Engineering Products Corporation, Aurora, Illinois, USA). Precisely, the samples were immersed in 50% RDO for the first 3 days and then transferred to 25% RDO for the remaining time. Fresh batch of the solution were prepared every 24 hours and substituted, according to a previously optimized protocol^87^.

The decalcification of the periotic bone was stopped when the vestibular scala and the *stria vascularis* of the cochlea were exposed. Subsequently, the cochlea was dissected, dehydrated with increasing concentrations of ethanol, critical point dried with CO_2_, and then coated with gold-palladium^88,89^. The sample was evaluated with a Hitachi S-4700 scanning electron microscope (SEM) at the University of British Columbia Bioimaging Facility for evidence of acoustic trauma.

### Parasitological investigations

The animals were surveyed for ectoparasites and endoparasites. The three stomach compartments were opened and washed on a sieve with 1 mm^2^ mesh to separate and collect helminths. Portions of the small and large intestine (10 meters/animal) were removed and the sediment, obtained after washing on sieve (1 mm^2^ mesh) and decantation on conical glass, were examined for parasites using a stereomicroscope. The stomach content, composed mainly of cephalopods beaks, was removed and stored for further prey identification. All the parasites recovered during *post-mortem* examination were washed and counted; most of them were stored in ethanol 70%, while anisakid nematodes were frozen at −80 °C. The morphometric identification was based on Oliver and Trilles^90^ methodology for crustacean parasites while for the collected helminths on Delyamure^91^ and on Mattiucci *et al*.^92^.

Genetic identification of the adults and pre-adults of *Anisakis* spp. collected during the parasitological survey was first undertaken using multilocus allozyme electrophoresis (MAE) on the frozen samples, according to the methodology reported in detail by Mattiucci *et al*.^92^. Furthermore, a subsample (N = 100) of the same specimens was sequenced at the mtDNA *cox2* gene, according to the procedures described by Valentini *et al*.^24^ and by Mattiucci *et al*.^25^.

Fecal samples were collected and immersed in a high-density solution (sodium nitrate and sodium thiosulphate/1.450) prior to microscopic examination. Stool smears were stained by a modified Ziehl Neelsen (MZN) technique to detect *Cryptosporidium* spp. Molecular analyses were performed using a PCR^93^ and a real-time PCR^94,95^.

Tissue samples were analyzed for the detection of *Toxoplasma gondii* using a PCR technique amplifying a conserved region of the nss-rRNA gene of Apicomplexa^96^. DNA extraction was performed on the tissues using NucleoSpin® Tissue kit (Macherey-Nagel, Germany). The reaction was carried out in 30 µl volume containing 1 X PCR buffer, 2 mM MgCl2, 200 µM each of the dNTPs, 2 U Platinum® Taq DNA Polymerase (Invitrogen, UK), 1 µM of each primer, and 1–3 µl of DNA extract. The reaction mixture was first treated at 95 °C for 5 min, followed by 12 cycles at 94 °C for 30 sec and 58 °C for 30 sec (decreasing each step −0.5 °C from 58 °C to 52 °C); 23 cycles at 94 °C for 30 sec, 52 °C for 30 sec and 72 °C for 30 sec and a final extension step at 72 °C for 7 min. DNA of *T*. *gondii* oocysts (GenBank^TM^ accession no. AY663792), isolated from a domestic cat, were used as positive control. PCR products were analyzed by electrophoresis in SYBR Safe stained (Invitrogen, UK) 2% agarose gel, visualized with Geldoc XR (Bio-Rad Laboratories, USA) under UV light, subsequently purified and sequenced at BMR-Genomics (Padova, Italy).

### Biotoxins

The gastric contents of the 3 sperm whales were analyzed for the presence of saxitoxins, domoic acid and lipophilic biotoxins (okadaic acid, dinophysistoxins, pectenotoxins, yessotoxins and azaspiracids).

The search for saxitoxins (SXTs) was carried out according to the AOAC method 959.08. This is a biological method based on an acid extraction of the toxins that is then inoculated in mice.

The search for domoic acid (DA) was carried out according to the European Reference Laboratory Method, EURL SOP for determination of domoic acid version June 1, 2008. DA was extracted by a mixture of methanol and water, then filtered through a membrane filter and analyzed by HPLC with UV detection.

The search for lipophilic toxins (DSP) was performed according to the European Reference Laboratory Method. This method was carried out for four chemical groups of toxins: okadaic acid (OA), including dinophysistoxins (DTX), pectenotoxins (PTX), azaspiracids (AZA) and yessotoxins (YTX). The method is based on the extraction of the toxins with 100% methanol from homogenized tissue. The extracts were filtered and analyzed by liquid chromatography coupled mass spectrometry. To determine the total content of OA group toxins, an alkaline hydrolysis is necessary from ethanolic extract to convert any acylated esters to the parent toxin.

### Analytical chemistry

Data regarding heavy metal analyses have been published separately^26,49^. In the present article, the results of metal analyses were compared with all findings herein reported, and considered in the Discussion section. Further, previously unreported, analyses were performed on tissues sampled during necropsies of the three whales, and detailed here below.

#### Analytical chemistry - lipid extraction

Blubber samples were freeze-dried and extracted with n-hexane in a Soxhlet apparatus. The lipid content of the extracted organic material was determined by gravimetry.

#### Analytical chemistry - organochlorines (OCs)

Analyses for HCB, DDTs and PCBs were performed according to methods recommended by the U.S. Environmental Protection Agency (EPA) 8081/8082 with modifications^97^. Tissue samples (about 2 g) were lyophilized in an Edwards freeze drier for 2 days, and extracted with n-hexane for gas chromatography (Merck) in a Soxhlet apparatus for analysis of organochlorine compounds. Whatman-H cellulose thimbles (i.d. 25 mm, e.d. 27 mm, length 100 mm) to be used for extraction of the samples were preheated for about 30 min at 110 °C and pre-extracted for 9 h in a Soxhlet apparatus with n-hexane, to remove organochlorine contamination. Each sample was spiked with surrogate compound (2,4,6-trichlorobiphenyls - IUPAC number 30)^98^ prior to extraction. This compound was quantified and its recovery calculated. Surrogate recovery was reported with the sample results. The samples were then purified with sulphuric acid to obtain a first lipid sedimentation. The extract then underwent liquid chromatography on a column containing Florisil that had been dried for 1 h in an oven at 110 °C. This further purified the apolar phase of lipids that could not be saponified, including steroids like cholesterol. Decachlorobiphenyl (DCBP - IUPAC number 209) was used as an internal standard, added to each sample extract prior to analysis, and included in the calibration standard, constituted by a mixture of specific compounds (Aroclor 1260, HCB and pp9- and op9-DDT, DDD and DDE). The analytical method used was High Resolution Capillary Gas Chromatography with an Agilent 6890 N and a 63Ni ECD and an SBP-5 bonded phase capillary column (30 m long, 0.2 mm i.d.). The carrier gas was nitrogen with a head pressure of 15.5 psi [ = 0.1068687 MPa] (splitting ratio 50/1). The scavenger gas was argon/methane (95/5) at 40 ml/min. Oven temperature was 100 °C for the first 10 min, after which it was increased to 280 °C at 5 °C/min. Injector and detector temperatures were 200 °C and 280 °C respectively. The extracted organic material (EOM%) from freeze-dried samples was calculated in all samples. Capillary gas chromatography revealed op9- and pp9- isomers of DDT and its derivatives DDD and DDE, and about 30 PCB congeners. Total PCBs were quantified as the sum of all congeners. These congeners constituted 80% of the total peak area of PCBs in the samples. Total DDT was calculated as the sum of op9DDT, pp9DDT, op9DDD, pp9DDD, op9DDE and pp9DDE. The results were expressed in ng/g lipidic weight (ng/g lipidic weight).

#### Analytical chemistry - Dioxins

The toxicologically relevant 7 Polychlordibenzo-p-dioxins (PCDD); the 10 Polychlordibenzofurans (PCDF) congeners; and the 12 dioxin-like polychlorbiphenyl congeners (DL-PCB), were investigated in the blubber of the three adult sperm whales and in the spermaceti of SW3. The overall toxicity of analytical products was calculated as Toxicity Equivalent Quantity (TEQ) by multiplying the effective concentration of each congener for the Factor of Toxicity Equivalent (TEF) to the toxicity value of 2,3,7,8-tetraclorodibenzodioxin, according to Van den Berg and colleagues^99^. The same specimens were analyzed also for the presence of the six non dioxin-like-polychlorbiphenyl (NLD-PCB) indicators (ICES-6), and their value was calculated as sum of the congeners. All values were expressed as “upper bound” concentration in pg/g whole weight (NL-PCBs in ng/g whole weight), so that values below of the limit of quantification (LOQ) were considered equal to the LOQ itself. All analyses were performed according to U.S. Environmental Protection Agency method 1613 rev. B, and method 1668 rev. B, respectively for Dioxins (PCDD and PCDF) and PCBs (LD-PCB and NLD-PCB), in-house modified to adapt the procedures to specific matrix^100,101^.

#### Statistical analysis

The non-parametric Kruskal-Wallis test was used to compare the levels in the biological matrixes of the three considered xenobiotics. The Kolmogorov-Smirnov test was employed to evaluate any significant difference (p < 0.1)^102^, as used in other studies on these species^12,53,63^.

#### Meteorological and stranding data sets

A series of multidisciplinary and wide range investigations were performed to assess any possible environmental cause temporally and spatially associated to the mass stranding. Weather and marine data from August 1, 2014 were obtained from different national and international meteorological archives, namely the Institute for the Protection of the Environment and Research (ISPRA, www.idromare.it), the Marine Forecasting Systems National Group of Operational Oceanography of the National Institute for Geophysics and Vulcanology (GNOO-INGV, http://gnoo.bo.ingv.it), the European Ocean Observatory Network (data from the observatory E2-M3A of the project EuroSITES, www. eurosites.info) and the “Poseidon System” of the Hellenic Center for Marine Research (www.poseidon.hcmr.gr). The INGV also provided information on past seaquakes and geomagnetic field abnormalities (www.earthquake.rm.ingv.it).

Since previous studies reported that solar activity and lunar cycles are among the natural factors that potentially cause and/or influence sperm whale mass stranding events, specific information was obtained from the US National Oceanic and Atmospheric Administration (NOAA, www.swpc.noaa.gov).

Any military activity or authorized seismic surveys in the surrounding waters were searched for in the governmental and official websites.

Finally, historical data sets on cetacean strandings along the Central and Southern Adriatic Sea coastline, and specifically those concerning sperm whales in Italy, were obtained from the Italian Cetacean Stranding Database (www.mammiferimarini.unipv.it/).

## Electronic supplementary material

Supplementary Dataset 1
