# Supplementary material for: Multidisciplinary studies on a sick-leader syndrome-associated mass stranding of sperm whales (Physeter macrocephalus) along the Adriatic coast of Italy
Source: Sci Rep. 2018 Aug 1;8:11577. doi: 10.1038/s41598-018-29966-7 (PMC6070578; doi:10.1038/s41598-018-29966-7)
Supplement: Supplementary file 1 — Supplementary Dataset 1 [file 41598_2018_29966_MOESM1_ESM.docx]

**Multidisciplinary studies on a sick-leader syndrome-associated mass stranding of sperm whales (*Physeter macrocephalus*) along the Adriatic coast of Italy**

Sandro Mazzariol^1^, Cinzia Centelleghe^1,*^, Bruno Cozzi^1^, Michele Povinelli^1^, Federica Marcer^2^, Nicola Ferri^3^, Gabriella Di Francesco^3^, Pietro Badagliacca^3^, Francesca Profeta^4^, Vincenzo Olivieri^5^, Sergio Guccione^5^, Cristiano Cocumelli^6^, Giuliana Terracciano^6^, Pasquale Troiano^7^, Matteo Beverelli^7^, Fulvio Garibaldi^8^, Michela Podestà^9^, Letizia Marsili^10^, Maria Cristina Fossi^10^, Simonetta Mattiucci^11^, Paolo Cipriani^11^, Daniele De Nurra^12^, Annalisa Zaccaroni^13^, Silva Rubini^14^, Daniela Berto^15^, Yara de Beraldo Quiros^16^, Antonio Fernandez^16^, Maria Morell^17^, Federica Giorda^18^, Alessandra Pautasso^18^, Paola Modesto^18^, Cristina Casalone^18,^ Giovanni Di Guardo^4^

1. Department of Comparative Biomedicine and Food Science, University of Padova, Padova, Italy
2. Department of Animal Medicine, Production and Health, University of Padova, Padova, Italy
3. Istituto Zooprofilattico Sperimentale dell’Abruzzo e del Molise “G. Caporale”, Teramo, Italy
4. University of Teramo, Faculty of Veterinary Medicine, Località Piano d'Accio, 64100 - Teramo, Italy
5. Centro Studi Cetacei, Pescara (CSC), Italy
6. Istituto Zooprofilattico Sperimentale del Lazio e della Toscana M. Aleandri, Rome, Italy
7. Istituto Zooprofilattico Sperimentale della Puglia e della Basilicata, Foggia, Italy
8. Department DISTAV, University of Genova, Genova, Italy
9. Museum of Natural History of Milan, Milano, Italy
10. Department of Physical Science, Earth and Environment, University of Siena, Siena, Italy
11. Department of Public Health and Infectious Diseases, University La Sapienza, Rome, Italy
12. Istituto Zooprofilattico Sperimentale della Sardegna, Sassari, Italy
13. Department of Veterinary Science, University of Bologna, Bologna, Italy
14. Istituto Zooprofilattico Sperimentale della Lombardia e dell’Emilia Romagna, Ferrara, Italy
15. ISPRA, Chioggia, Italy
16. Institute of Animal Health and Food Safety, Universitad de Las Palmas de Gran Canaria, Las Palmas, Spain
17. Institute for Neurosciences of Montpellier (Inserm UMR 1051), Montpellier, France
18. Istituto Zooprofilattico Sperimentale del Piemonte, Liguria e Val d’Aosta, Torino, Italy

*: Corresponding author: Cinzia Centelleghe, cinzia.centelleghe@gmail.com

Viale dell’Università 16, 35020 Legnaro (PD)

**Supplementary Figure S1. FCA analyses implemented by using GENETIX Software.** Genotypes are clusterized in 2 different groups, BLUE including samples from the sperm whales stranded in Vasto, and YELLOW reporting samples provide by MMMTB of the University di Padova.


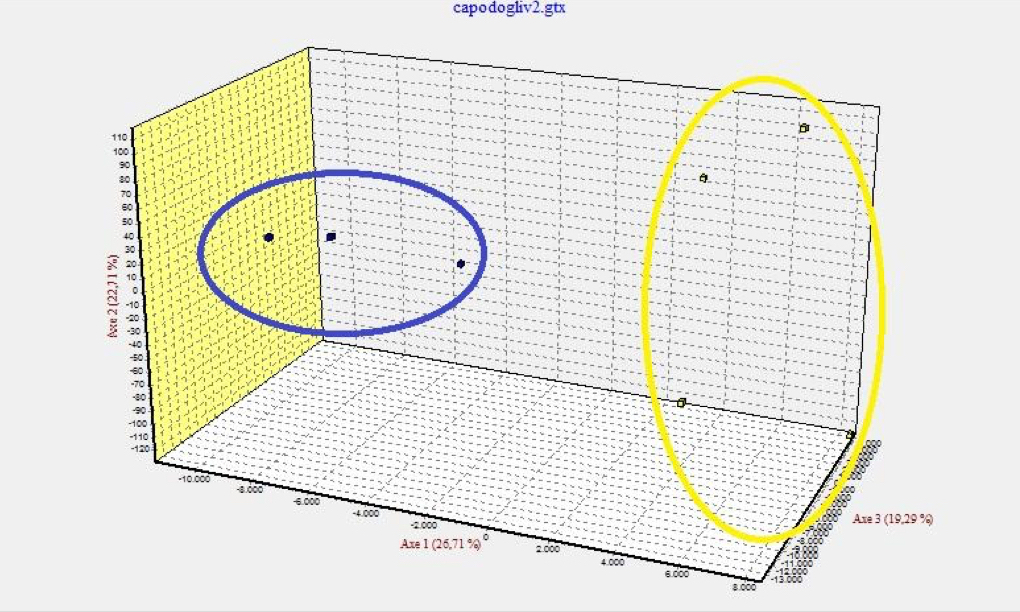


**Supplemantary Table S2.** Concentrations of all the considered OCs (HCB, DDTs e PCBs) found in tissues from the 4 sperm wahles expressed in ng/g on lipidic base; lipid extracted (%) and rate among different compound are also reported.

| **BLUBBER** | **SW1** | **SW2** | **SW3** | **SW1b** |
| --- | --- | --- | --- | --- |
| **Lipidic weight%** | 76,60 | 68,40 | 68,60 | 35,10 |
| **HCB (ng/g l.b.)** | 183,35 | 132,71 | 220,66 | 61,68 |
| **DDTs (ng/g l.b.)** | 34357,22 | 11549,62 | 32727,55 | 22229,68 |
| **PCBs (ng/g l.b.)** | 22722,59 | 9516,10 | 14409,49 | 15369,42 |
| **pp'DDE/DDTs** | 0,81 | 0,78 | 0,83 | 0,84 |
| **pp'DDE/pp'DDT** | 11,78 | 10,79 | 16,14 | 20,74 |
| **DDTs/PCBs** | 1,51 | 1,21 | 2,27 | 1,45 |
| **MUSCLE** | **SW1** | **SW2** | **SW3** | **SW1b** |
| **Lipidic weight%** | 3,30 | 3,70 | 3,20 | 14,70 |
| **HCB (ng/g l.b.)** | 107,70 | 52,77 | 15,10 | 29,94 |
| **DDTs (ng/g l.b.)** | 47800,13 | 16048,15 | 1717,17 | 6861,90 |
| **PCBs (ng/g l.b.)** | 26570,28 | 15919,01 | 2779,43 | 4646,65 |
| **pp'DDE/DDTs** | 0,82 | 0,81 | 0,85 | 0,83 |
| **pp'DDE/pp'DDT** | 14,63 | 13,52 | 23,15 | 20,72 |
| **DDTs/PCBs** | 1,80 | 1,01 | 0,62 | 1,48 |
| **LIVER** | **SW1** | **SW2** | **SW3** | **SW1b** |
| **Lipidic weight%** | 9,80 | 11,46 | 20,92 | 9,90 |
| **HCB (ng/g l.b.)** | 25,94 | 11,38 | 29,81 | 12,16 |
| **DDTs (ng/g l.b.)** | 9576,16 | 4634,24 | 12714,76 | 3376,26 |
| **PCBs (ng/g l.b.)** | 7771,74 | 4280,01 | 5589,12 | 2650,95 |
| **pp'DDE/DDTs** | 0,84 | 0,85 | 0,86 | 0,84 |
| **pp'DDE/pp'DDT** | 23,81 | 46,05 | 44,82 | 45,90 |
| **DDTs/PCBs** | 1,23 | 1,08 | 2,28 | 1,27 |
| **BRAIN** | **SW1** | **SW2** | **SW3** | **SW1b** |
| **Lipidic weight%** | n.e. | 24,70 | 49,70 | n.e. |
| **HCB (ng/g l.b.)** | n.e. | 9,25 | 4,84 | n.e. |
| **DDTs (ng/g l.b.)** | n.e. | 1328,82 | 1105,73 | n.e. |
| **PCBs (ng/g l.b.)** | n.e. | 1361,63 | 787,31 | n.e. |
| **pp'DDE/DDTs** | n.e. | 0,81 | 0,82 | n.e. |
| **pp'DDE/pp'DDT** | n.e. | 0,98 | 1,40 | n.e. |
| **DDTs/PCBs** | n.e. | 25,93 | 23,17 | n.e. |

**Supplemantary Table S3.** Average concentrations of PBDEs found in tissues sampled from SW1, SW1b, SW2 and SW3.

| **BLUBBER** | **SW 1** | **SW1b** | **SW 2** | **SW 3** |
| --- | --- | --- | --- | --- |
| **BDE 28** | 2,40 | 2,16 | 2,05 | n.q |
| **BDE 47** | 78,8 | 38,5 | 65,1 | 99,8 |
| **BDE 99** | 20,3 | 16,2 | 24,1 | 29,7 |
| **BDE 100** | 27,7 | 15,7 | 26,8 | 32,0 |
| **BDE 153** |  | 4,55 |  |  |
| **BDE 154** | 31,2 | 25,5 | 40,3 | 21,5 |
| **BDE 209** |  | 2,47 |  | 0,08 |
| **ΣPBDEs** | 160 | 105 | 158 | 183 |
| **MUSCLE** | **SW 1** | **SW1b** | **SW 2** | **SW 3** |
| **BDE 28** |  |  |  |  |
| **BDE 47** | 15,5 | 57,2 | 59,9 | 71,6 |
| **BDE 99** |  | 34,3 |  |  |
| **BDE 100** |  |  |  |  |
| **BDE 153** |  |  |  |  |
| **BDE 154** |  |  |  |  |
| **BDE 209** |  |  | 12,8 | 8,11 |
| **ΣPBDEs** | 15,5 | 91,4 | 72,2 | 79,7 |
| **HEART** | **SW 1** | **SW1b** | **SW 2** | **SW 3** |
| **BDE 28** |  |  |  |  |
| **BDE 47** | 40,5 | 32,1 | 46,1 | 83,5 |
| **BDE 99** |  |  | 22,0 | 23,7 |
| **BDE 100** |  | 9,14 |  | 28,5 |
| **BDE 153** |  |  |  |  |
| **BDE 154** | 17,5 |  | 17,3 |  |
| **BDE 209** | 3,29 | 4,10 |  |  |
| **ΣPBDEs** | 61,3 | 45,4 | 85,4 | 138 |
| **LIVER** | **SW 1** | **SW1b** | **SW 2** | **SW 3** |
| **BDE 28** |  | nd |  |  |
| **BDE 47** |  | nd |  |  |
| **BDE 99** | 23,8 | nd |  | 14,8 |
| **BDE 100** |  | nd |  | 17,8 |
| **BDE 153** |  | nd | 89,6 | 4,65 |
| **BDE 154** | 27,5 | nd | 40,9 | 33,9 |
| **BDE 209** |  | nd | 6,12 | 22,4 |
| **ΣPBDEs** | 51,3 | nd | 137 | 93,5 |
| **BRAIN** | **SW 1** | **SW1b** | **SW 2** | **SW 3** |
| **BDE 28** |  | nd |  | nd |
| **BDE 47** | 15,9 | nd | 7,26 | nd |
| **BDE 99** |  | nd |  | nd |
| **BDE 100** |  | nd |  | nd |
| **BDE 153** |  | nd |  | nd |
| **BDE 154** |  | nd | 3,27 | nd |
| **BDE 209** | 0,20 | nd |  | nd |
| **ΣPBDEs** | 16,1 | nd | 10,5 | nd |
